# Prediction of Both E-Jet Printing Ejection Cycle Time and Droplet Diameter Based on Random Forest Regression

**DOI:** 10.3390/mi14030623

**Published:** 2023-03-08

**Authors:** Yuanfen Chen, Zongkun Lao, Renzhi Wang, Jinwei Li, Jingyao Gai, Hui You

**Affiliations:** School of Mechanical Engineering, Guangxi University, Nanning 530004, China

**Keywords:** e-jet printing, ejection cycle time prediction, droplet diameter prediction, random forest regression

## Abstract

Electrohydrodynamic jet (E-jet) printing has broad application prospects in the preparation of flexible electronics and optical devices. Ejection cycle time and droplet size are two key factors affecting E-jet-printing quality, but due to the complex process of E-jet printing, it remains a challenge to establish accurate relationships among ejection cycle time and droplet diameter and printing parameters. This paper develops a model based on random forest regression (RFR) for E-jet-printing prediction. Trained with 72 groups of experimental data obtained under four printing parameters (voltage, nozzle-to-substrate distance, liquid viscosity, and liquid conductivity), the RFR model achieved a MAPE (mean absolute percent error) of 4.35% and an RMSE (root mean square error) of 0.04 ms for eject cycle prediction, as well as a MAPE of 2.89% and an RMSE of 0.96 μm for droplet diameter prediction. With limited training data, the RFR model achieved the best prediction accuracy among several machine-learning models (RFR, CART, SVR, and ANN). The proposed prediction model provides an efficient and effective way to simultaneously predict the ejection cycle time and droplet diameter, advancing E-jet printing toward the goal of accurate, drop-on-demand printing.

## 1. Introduction

Electrohydrodynamic jet (E-jet) printing is a new additive manufacturing technology in which a fine solution jet is ejected from the tip of a Taylor cone under the action of an electric field [1] and deposited on a substrate to form a micro-dot pattern. Due to the advantages of direct writing, noncontact, high resolution, and drop-on-demand, E-jet printing has been applied in biological 3D structures, cell-laden microspheres [2,3], light-emitting diodes [4,5], transistors [6,7], transparent electrodes [8,9], optical micro-lenses [10], sensors [11,12,13], flexible electronic devices [14,15,16], and other fields. Under constant DC voltage, the ejection cycle time and droplet diameter, together with the substrate moving speed, determine the quality of an E-jet printing pattern. The prediction and control of these three key parameters precisely are important to achieve on-demand printing. Among them, the substrate moving speed can be directly controlled, while the ejection cycle time and droplet diameter are affected by a variety of process parameters, such as liquid properties, voltage magnitude, supply flow rate, and nozzle-to-substrate distance. Due to the complex process of E-jet printing, which involves several coupled physical fields, predicting and controlling the ejection cycle time and droplet diameter, especially simultaneously prediction, remain a challenge. For printing under a pulse voltage, each printing cycle consists of one or more DC ejection cycle, depending on the pulse width. Thus, studying cycle time and droplet size under DC voltage is an important guide for on-demand printing under a pulse voltage.

Researchers all over the world have conducted a variety of research to study the E-jet-printing process, and have reported some formulas and prediction models to predict either droplet ejection or droplet diameter. Ejection cycle time has been predicted by formulas that relate the ejection frequency to different printing parameters. An et al. [17] studied the relationships between ejection frequency and liquid viscosity, conductivity, and surface tension and then proposed a theoretical formula for ejection frequency estimation. Chen et al. [18] proposed a scaling law of ejection frequency with process parameters (electric field, viscosity, and conductivity). Choi et al. [19] obtained the relationship between ejection frequency and the electric field but ignored the effects of liquid viscosity and conductivity. Bober et al. [20] developed a theoretical prediction equation relating ejection frequency with liquid supply flow rate. Ball et al. [21] predicted ejection frequency with an improved firefly algorithm. Unlike ejection cycle time, droplet diameter is usually predicted by simulation models. Qian et al. [22] researched the effect of voltage magnitude and air pressure on the droplet diameter of E-jet printing and predicted the droplet diameter by theoretical modeling. Wang et al. [23] derived a simplified model for droplet diameter prediction based on the balance of electric field, surface tension, and gravity. Collins et al. [24] explained the mechanism of Taylor cone formation, jet ejection, and droplet breakup by numerical simulation and then proposed a scaling law for droplet diameter prediction. Jiang et al. [25] used CFD modeling to simulate the whole process of E-jet printing and predicted droplet diameter under different process parameters. Guo et al. [26] used a polynomial regression analysis to establish a model predicting droplet volume under several parameters (voltage, nozzle-to-substrate distance, fluid viscosity, and fluid conductivity). These experimental research projects and theoretical modeling studies have provided effective ways to predict ejection cycle time and droplet diameter under certain printing conditions, but none of them can simultaneously predict ejection cycle time and droplet diameter. As the key factors that affect the quality of a desired printed pattern, ejection cycle time and droplet diameter should be controlled simultaneously in an on-demand E-jet-printing process. A method that can accurately and economically predict ejection cycle time and droplet diameter simultaneously is required.

In recent years, machine-learning algorithms have been widely used as a data-driven modeling method in many fields, such as geotechnical engineering [27], traffic safety [28], material engineering [29], and biomedicine [30]. To improve computational accuracy and computational efficiency, Breima proposed the random forest (RF) algorithm in 2001 [31]. Since then, RF has been successfully applied to many fields, such as power system load prediction [32], genetic engineering [33], and chemical engineering [34], for its high prediction precision, fast calculation speed, decreased overfitting, and strong generalization ability.

To address the challenge of E-jet printing ejection cycle time and droplet diameter prediction, this paper proposes a model based on a RFR algorithm to predict ejection cycle time and droplet diameter simultaneously. Voltage and nozzle-to-substrate distance can be adjusted easily during the E-jet-printing process, while viscosity and conductivity can be adjusted on demand during ink preparation. Thus, voltage magnitude, nozzle-to-substrate distance, liquid viscosity, and liquid conductivity are selected for the full factorial experimental design of experimental printing data. Then, an RFR prediction model is established, trained with 72 groups of experimental data, and applied to predict ejection cycle time and droplet diameter simultaneously. The prediction accuracy of the RFR is compared with a classification and regression tree (CART), support vector regression (SVR), and an artificial neural network (ANN). The results show that, with limited training data, the RFR has the best prediction performance, proving the precision and suitability of the RFR for E-jet prediction. The proposed prediction model enables fast, economical, accurate prediction of both ejection cycle time and droplet diameter, guiding further optimization of the E-jet-printing process.

## 2. Materials and Methods

### 2.1. E-Jet-Printing System

**Figure 1:**
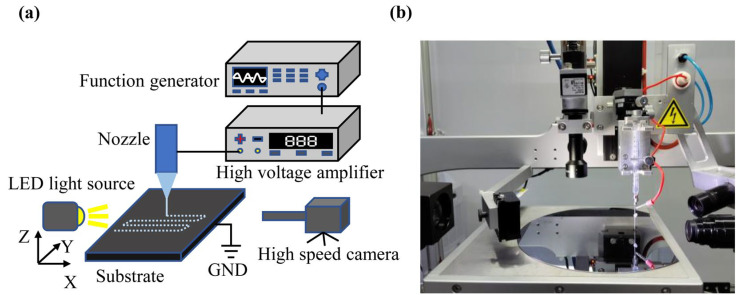
(**a**) Schematic diagram of E-jet-printing system; (**b**) picture of E-jet-printing system.

A schematic diagram and a picture of the E-jet-printing system are shown in Figure 1. In addition, a picture of the printing nozzle is shown in Figure S1 in the Supporting Materials.

The E-jet-printing system was mainly composed of a liquid supply module (liquid reservoir tube and printing nozzle), a voltage module (function generator and high-voltage amplifier), a motion module (mobile *Z*-axis and XY moving stage), an observation module (high-speed camera and LED light source), and a control module (computer).

A printing nozzle with an outer diameter of 50 um was prepared with a micropipette puller (Sutter P-1000, Sutter Instrument Company, Novato, CA, USA). The printing nozzle was connected to a liquid reservoir tube that was fixed to the *Z*-axis. To avoid the impact of air pressure fluctuations on the experimental results, no back pressure was added to the liquid reservoir tube, and the liquid was delivered simply by the capillary action of the thin nozzle. The distance between the nozzle and the substrate (silicon wafer, 200 mm in diameter and 800 µm thick) was controlled by moving the nozzle up and down through a precision motor and then measured via real-time imaging. The applied voltage was generated by a function generator (Keysight-33500B Series, Keysight Technologies Co., Ltd., Santa Rosa, CA, USA) and a high-voltage amplifier (HVA-103NP6, Tianjin Shenghuo Technology Co., Ltd., Tianjin, China) with an output voltage range of 0 ~±5 KV. The high-voltage amplifier was connected to the nozzle, and the ground was connected to the substrate, forming an electric field between the nozzle and the substrate. Under the action of constant DC voltage, the liquid formed a Taylor cone at the tip of the nozzle and ejected a fine jet with a certain cycle. The droplet ejection process was recorded with a high-speed camera (Fastcam Mini UX 100, photron, Kyoto, Japan), and the droplet ejection cycle time was calculated based on the high-speed images. The substrate was fixed to the XY stage through vacuum adsorption to collect the deposited droplets. Droplet diameter was measured with a metallographic microscope (53X-V, Shanghai Optics Instrument Five Co., Ltd., Shanghai, China). 

### 2.2. E-Jet-Printing Liquids

The printing liquids were a mixture of glycerol, deionized water, and aqueous sodium chloride (NaCl). The NaCl aqueous solution was prepared by dissolving 0.2 g NaCl into 40 mL deionized water. The conductivity of the printing liquids could be adjusted by changing the volume ratio of the NaCl solution, and the viscosity could be adjusted by changing the volume ratio of the glycerol. The viscosity, conductivity, surface tension, and contact angle of the printing liquid were measured with a viscometer (SHCP1, Guangzhou Leadtek Instrument Technology Co., Ltd., Guangzhou, China), a conductivity meter (DZS-708 L, Shanghai Yidian Scientific Instruments Co., Ltd., Shanghai, China), and a contact angle meter (SDC-200S, Dongguan Shengding Precision Instrument Co., Ltd., Dongguan, China), respectively. The properties of the printing liquid are shown in Table 1. The corresponding compositions of the printing liquids used in the experiments are shown in Table S1.

### 2.3. Printing Experimental Design

For the printing experiment, four parameters affecting E-jet printing (voltage, nozzle-to-substrate distance, liquid viscosity, and liquid conductivity) [26] were selected as the variables. Each variable had 3 levels, forming 81 sets of experimental results, as shown in Table 2. Through experimental research, it was found that, in order to keep the printing in pulse jet mode, the range of the electric field strength could only vary in a small range. If the electric field strength was too low, no Taylor cone was formed; if the electric field strength was too high, a continuous jet was produced. However, the small change in electric field strength still had an important impact on the process of jet printing, as shown in Figures S2 and S3. When the nozzle-to-substrate distance was constant, as voltage increased from 1150 V to 1170 V, the ejection cycle time decreased from 2.06 ms to 1.23 ms, decreasing 67.5%. When the voltage was constant, and the nozzle-to-substrate distance increased from 280 μm to 300 μm, the ejection cycle time increased from 1.10 ms to 2.06 ms, increasing 87.3%.

## 3. Machine-Learning Methods

### 3.1. RFR Model

**Figure 2:**
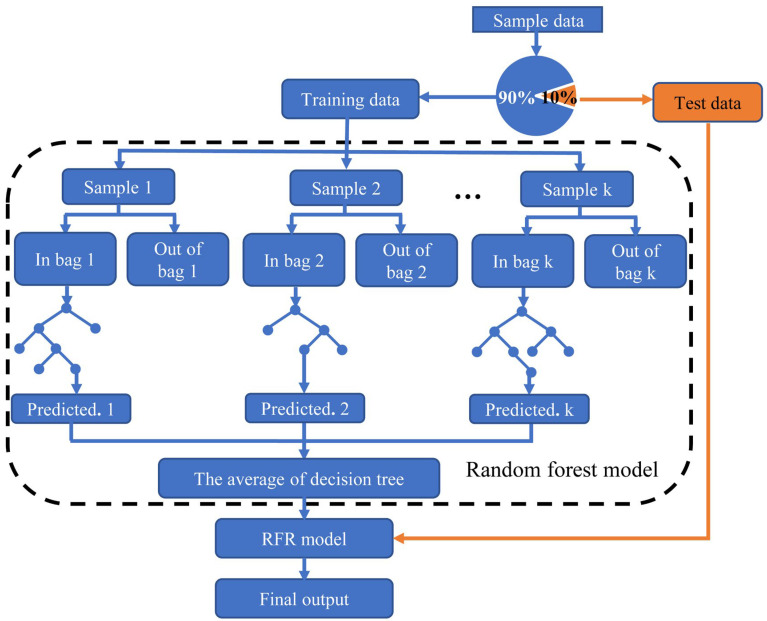
Flow chart of the RFR prediction model.

As an ensemble algorithm, random forest is based on decision trees and bagging sampling. A decision tree is a tree structure in which each internal node represents a judgment on an attribute, and each branch represents the output of a judgment result. Bagging sampling reduces generalization errors by combining multiple decision tree models. The principle is to train several different decision tree models independently. For regression problems, the mean of the decision tree is calculated as the final result. Random forest builds bagging ensembles with decision trees as base learners. Bootstrap technology is the sampling of samples with return. Random forest uses bootstrap technology to repeatedly and randomly select multiple samples and their features from sample sets with replacements and constructs multiple decision trees [35]. For regression problems, after a prediction model is established, an RFR prediction result is determined using the mean value of each decision tree. According to the above random forest principle, the flow chart of the RFR model for ejection cycle time and droplet diameter prediction is shown in Figure 2.

(1) Data reading and preprocessing: The E-jet printing experimental ejection cycle time result is read by the RFR model, divided into a training set and a test set in a ratio of 9:1, and then preprocessed to ensure all data obey the standard normal distribution. The same process is repeated for the experimental droplet diameter results.

(2) Sample set generation: The bootstrap is used to extract a sample set with the same capacity from the training set, which is repeated for k times to obtain k sample sets: $\theta_{1}$, $\theta_{2}$,…, $\theta_{k}$. In particular, when k = 1, the model is a CART.

(3) Decision tree generation: E-jet printing ejection cycle time and droplet diameter prediction is a regression problem. For the regression problem, the basic learner of the random forest is a CART, and its nodes are split according to the principle of minimum mean squared error (MSE), as shown in Equation (1).

(1) $$\underset{A,s}{\underset{︸}{\min}}\left[\underset{c_{l}}{\underset{︸}{\min}}\sum_{x_{i}\in D_{l}\left(A,s\right)}{\left(y_{i}-c_{l}\right)}^{2}+\underset{c_{r}}{\underset{︸}{\min}}\sum_{x_{i}\in D_{r}\left(A,s\right)}{\left(y_{i}-c_{r}\right)}^{2}\right]$$

In Equation (1), $x_{i}$ is an n-dimensional input vector; $y_{i}$ is the output value; A is an arbitrary division of features; s is the division point of the corresponding feature; $D_{l}$ and $D_{r}$ are the left and right data sets of the sample set divided by s points, respectively; $c_{l}$ is the average output value of $D_{l}$; and $c_{r}$ is the average output value of $D_{r}$.

Each child node of the decision tree is continuously divided using Equation (1) until the set threshold is satisfied, dividing the sample set into M data sets: $D_{1}$, $D_{2}$,…$D_{m}$.

(2) $$T\left(x,\theta_{k}\right)=\sum_{m=1}^{M}\overset{\wedge }{c_{m}}I\left(x\in D_{m}\right)$$

(3) $$I=\left\{\begin{array}{c} 1,if\left(x\in D_{m}\right)\\ 0,if\left(x\notin D_{m}\right) \end{array}\right.$$

In Equations (2) and (3), I is the indicator function, and $\overset{\wedge }{c_{m}}$ is the mean value of the output of the data set $D_{m}$. Then k CARTs are generated using the sample set and random forest model, as shown in Equation (4).

(4) $$\left\{\left.T_{1}\left(x,\theta_{k}\right),T_{2}\left(x,\theta_{k}\right),\cdot \cdot \cdot ,T_{k}\left(x,\theta_{k}\right)\right\}\right.$$

For CART generation, the m-dimensional (m≤4) E-jet parameters are extracted without put-back from the above four key parameters (viscosity, conductivity, voltage, and nozzle-to-substrate distance) as the feature variables of each split node.

(4) Printing result prediction: The testing set S is input into the RFR model and all regression decision trees are traversed to obtain the prediction results. Then, the arithmetic mean of the regression decision trees is calculated as the final prediction result, as shown in Equation (5).

(5) $$F\left(x\right)=\frac{1}{k}\sum_{k=1}^{k}\left\{\left.T\left(x,\theta_{k}\right)\right\}\right.$$

### 3.2. SVR and ANN Model

**Figure 3:**
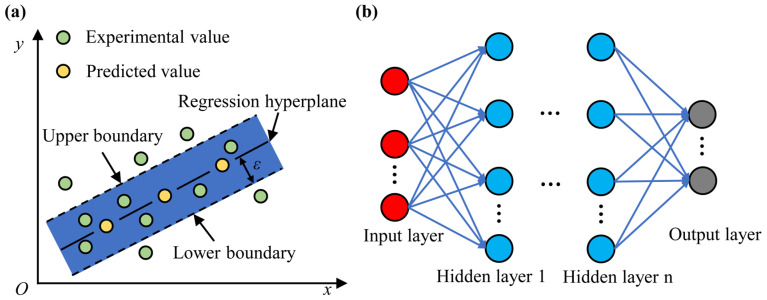
Schematic diagrams of machine-learning prediction models: (**a**) SVR; (**b**) ANN.

To further verify the applicability and accuracy of the RFR model with limited training data, other commonly applied machine-learning methods, SVR and ANN, were applied to predict ejection cycle time and droplet diameter. Schematic diagrams of SVR and ANN are shown in Figure 3.

The principle of SVR is shown in Figure 3a. Here, the most widely used Gaussian radial basis function (RBF) was selected as the kernel function of SVR. The values of the SVR internal parameters (*C*, *σ*, *ε*) determined the predictive performance of the model. *C* was the penalty factor in the SVR model, affecting the complexity and learning ability of the model, as well as the degree of approximation error. *σ* was the coefficient of the RBF kernel function, and it related to the radial range of the RBF kernel function. *ε* was the error tolerance of the SVR model, controlling the upper and lower boundaries in the SVR. The SVR model assumed that a maximum error of *ε* between the predict value and the experimental value could be tolerated (values within the lower and upper boundaries), and the loss was calculated only when the absolute value of the difference was greater than *ε*. In the SVR model, the experimental data were divided into a training set and a test set in a ratio of 9:1 and preprocessed to ensure all data obeyed the standard normal distribution. To find the optimal SVR model, the parameters (*C*, *σ*, *ε*) were then adjusted using a cross-validation grid search. The parameter combinations are shown in Table 3. Through the parameter search, the established SVR model had best prediction performance when *C* was 5, *σ* was 0.3, and *ε* was 0.01.

The ANN was mainly composed of an input cell, a hidden cell, and an output cell, as shown in Figure 3b. The number of hidden cells and neurons was related to the ANN prediction performance. In the ANN, the experimental data were divided into a training set and a test set in a ratio of 9:1 and then preprocessed to ensure all data obeyed the standard normal distribution. Due to the small amount of data in this paper, in order make the model converge faster, the lbfgs optimizer based on the quasi-Newton algorithm was selected. To reduce the computational cost, the number of hidden layers was selected as 3, and the relu function was selected as the activation function of the hidden cell. The number of hidden cell neurons in the ANN model was optimized using a cross-validation grid search, and the combination of search parameters is shown in Table 4. According to the search results, when the numbers of neurons in the 3 hidden cells were 5, 15, and 5, respectively, the ANN model had the best prediction performance.

### 3.3. Model Performance Evaluation

The mean absolute percent error (MAPE) and root mean square error (RMSE) were selected as the evaluation indices. The MAPE expression is shown in Equation (6). It could quantitatively reflect the accuracy of the prediction model. The smaller its value, the higher the prediction accuracy. The RMSE expression is shown in Equation (7). It was a measure of the deviation between the experimental value and the predicted value, reflecting the degree of dispersion of the sample.

(6) $$MAPE=\frac{1}{n}\sum_{i=1}^{n}\left|\frac{y_{i}-\overset{\wedge }{y_{i}}}{y_{i}}\right|\times 100\%$$

(7) $$RMSE=\sqrt{\frac{1}{n}\sum_{i=1}^{n}{\left|y_{i}-\overset{\wedge }{y_{i}}\right|}^{2}}$$

In Equations (6) and (7), n is the number of predicted samples; $y_{i}$ is the experimental value; and $\overset{\wedge }{y_{i}}$ is the model-predicted value.

## 4. Results and Discussions

### 4.1. Printing Results

**Figure 4:**
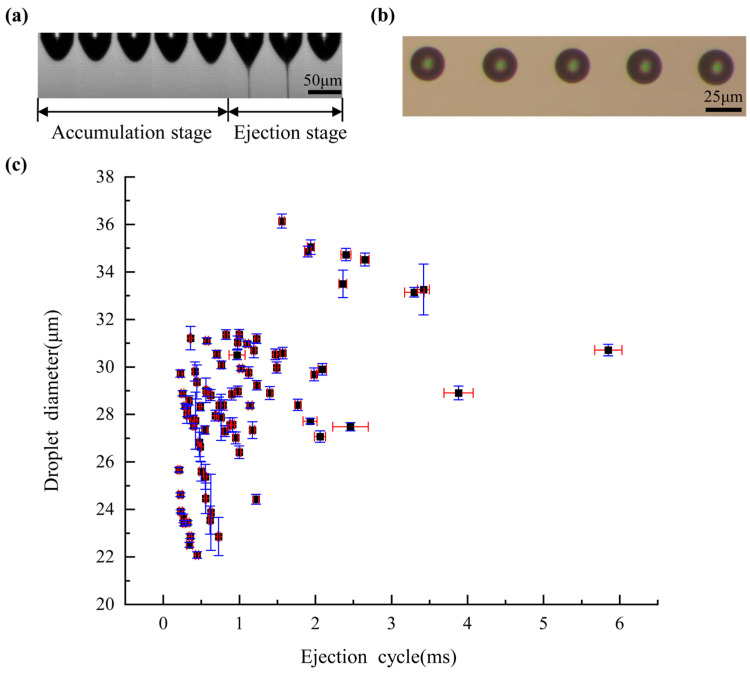
Printing results: (**a**) droplet ejection process; (**b**) microscope image of droplet on substrate; (**c**) ejection cycle time and droplet diameter data.

Precise prediction and control of ejection cycle time and droplet diameter is the key to ensure efficient and high-quality E-jet printing. Here, we proposed a RFR prediction model that was trained with limited experimental data sets. The experimental data of ejection cycle time and droplet diameter were obtained according to Table 2. The results are shown in Figure 4.

Under constant DC voltage, the liquid at the nozzle tip formed a Taylor cone and ejected a fine jet with a fixed cycle. A droplet formed on the moving substrate for each ejection cycle, and the distance between the droplets was determined by the ejection cycle time and substrate moving speed. In real experiments, it takes a lot of time to adjust the moving speed to control the droplet distance since the ejection cycle is difficult to predict and control. An accurate and efficient prediction model would save a lot of time. Here, the process of droplet ejection was recorded and measured with a high-speed camera, and a typical droplet ejection cycle is shown in Figure 4a. A complete droplet ejection process consisted of two stages: droplet accumulation and droplet ejection. During the droplet accumulation stage, the curved lunar surface was gradually transformed into a Taylor cone under the electric field force; during the droplet ejection stage, a fine jet was ejected from the tip of the Taylor cone and lasted for a certain period of time.

The key factor determining the resolution of a printing pattern is the size of the droplet diameter. The droplet diameter was measured with a metallographic microscope, and typical, on-demand E-jet-printing droplets are shown in Figure 4b. The droplet diameter was mainly determined by the volume of the jetted fluid, as well as by the contact angle between the fluid and the substrate. The volume of the jetted fluid depended on the voltage magnitude, nozzle-to-substrate distance, surface tension of the liquid, liquid conductivity, and liquid viscosity. The contact angle depended on the surface tension of the liquid and the surface properties of the substrate. All the experiments in this paper used the same silicon substrate, so the droplet diameter was mainly determined by the process parameters of E-jet printing.

To improve the reliability of the prediction model, each group of printing experiments was repeated 10 times, and the mean value was selected as the final experimental result. The degree of variation of the experimental data was analyzed by the coefficient of variation (CV) (the ratio of the standard deviation to the mean value). The statistical results of the 81 groups of ejection cycle time and droplet diameter are shown in Figure 4c. The minimum CV for the ejection cycle time was 1.13%, and the maximum CV was 14.64%. In addition, 96.3% of the ejection cycle time results had a CV within 10%, indicating that the experimental results were highly reliable. The minimum CV was 0.29%, and the maximum CV was 6.70%. All the droplet diameter results had a CV within 10%, indicating that the experimental results were highly reliable. The reason for the variation in the CV could be because, in the experimental process, the E-jet-printing process is also affected by humidity, temperature, air cleanliness, and other unstable environmental factors.

### 4.2. Prediction Performance of RFR Model

**Figure 5:**
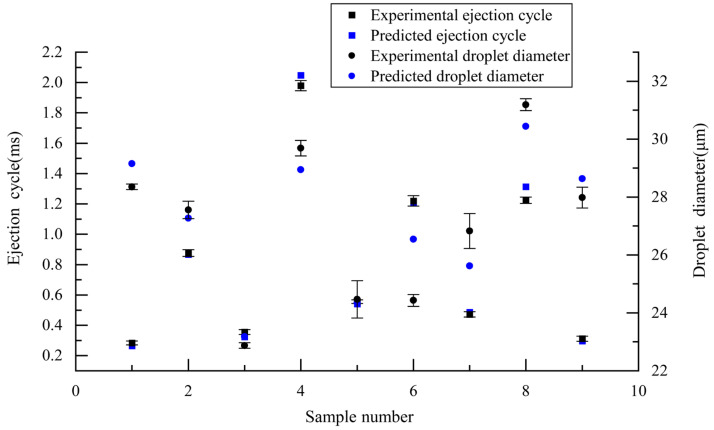
RFR prediction performance.

In the E-jet-printing process, ejection cycle time and droplet diameter size are the main factors that affect the quality of a printed pattern. Thus, the simultaneous prediction of these factors is beneficial to achieve efficient, high-quality, on-demand E-jet printing. Here, we developed an RFR model to achieve the simultaneous prediction of ejection cycle time and droplet diameter. To improve the accuracy of the RFR model, the value of the regression decision tree k was optimized to be 30 using the grid search method, and the RFR prediction results are shown in Figure 5 and Table 5.

It can be seen from Figure 5 that most of the predicted ejection cycle times were within the standard deviation of the experimental results, and most of the predicted droplet diameters were close to the experimental results. According to Table 5, the maximum relative error between the experimental value and the predicted value was −8.732% for ejection cycle time, and the maximum relative error between the experimental value and the predicted value was 8.628% for droplet diameter. Both were lower than 10%, indicating high accuracy of the RFR prediction model. For ejection cycle time prediction, the MAPE was 4.35% and the RMSE was 0.04 ms. For droplet diameter prediction, the MAPE was 2.89% and the RMSE was 0.96 μm. The prediction results show that the RFR model could accurately predict both the ejection cycle time and the droplet diameter with a small training data set. The proposed RFR model was an accurate and economical way to simultaneously predict the ejection cycle time and the droplet diameter.

### 4.3. Performance Comparison between Different Models

**Figure 6:**
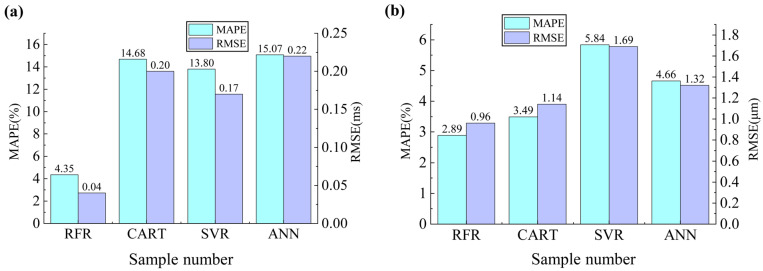
Performance comparison among different models: (**a**) prediction performances of ejection cycle time; (**b**) prediction performances of droplet diameter.

To further verify the effectiveness of the RFR, the prediction effect of the RFR was compared with CART, SVR, and ANN models. The predicted values of each model are shown in Tables S2 and S3 in the Supporting Material. The evaluation indices of each prediction model are shown in Figure 6. It can be seen that the RFR had the smallest MAPE and RMSE for both ejection cycle time and droplet diameter, indicating that the RFR had the best prediction performance. Ejection cycle time prediction performance was in the following order: RFR > SVR > CART > ANN. The SVR ranked second, but still its MAPE and RMSE were much higher than those of the RFR. In comparison, SVR training was slower, and the optimized model was more sensitive to parameter combination, while RFR training was faster and model optimization was easier to implement. Droplet diameter prediction performance was in the following order: RFR > CART > ANN > SVR. The CART ranked second, but still the MAPE and RMSE were much higher than those of the RFR. This was because the CART had a weak generalization ability and was prone to overfitting, while the RFR used the mean values of multiple regression decision trees as the final output, making the RFR more generalized and less prone to overfitting. The ANN had the worst overall prediction performance, mainly because the ANN required a large amount of experimental data for training, while the samples of E-jet printing ejection cycle time and droplet diameter were relatively small. In summary, for both ejection cycle time and droplet diameter prediction, the RFR was faster to train, easier to achieve model optimization, had higher generalization ability, was less prone to overfitting, and could achieve higher accuracy with limited experimental samples.

## 5. Conclusions

To address the problem of the simultaneous prediction of ejection cycle time and droplet diameter for E-jet printing, this paper proposed an RFR prediction model. Based on an E-jet-printing mechanism, four E-jet-printing process parameters (voltage, nozzle-to-substrate distance, liquid viscosity, and liquid conductivity) were selected as input features, and ejection cycle time and droplet diameter were taken as outputs to develop the RFR prediction model. The experimental and predicted results under different parameter combinations were obtained. With only 72 groups of training data, the developed RFR prediction model achieved a MAPE of 4.35% and an RMSE of 0.04 ms for ejection cycle time prediction, as well as a MAPE of 2.89% and an RMSE of 0.96 μm for droplet diameter prediction. The prediction results of the RFR were then compared with CART, SVR, and ANN models, showing that, with limited training data, the RFR had the best prediction performance. The proposed RFR prediction model could achieve the simultaneous and accurate prediction of ejection cycle time and droplet diameter, providing a simple, feasible, efficient method for on-demand E-jet-printing optimization.

## Figures and Tables

**Table 1:** Properties of the E-jet-printing liquids.

Number	Density (Kg/m^3^)	Conductivity (μS/cm)	Surface Tension(mN/m)	Viscosity(mPa·s)
1	1223.9	5	66.7	35
2	1223.9	10	66.7	35
3	1223.9	15	66.7	35
4	1231.6	5	67.3	50
5	1231.6	10	67.3	50
6	1231.6	15	67.3	50
7	1238.9	5	66.9	65
8	1238.9	10	66.9	65
9	1238.9	15	66.9	65

**Table 2:** Selected E-jet process parameters and levels.

Process Parameter	Level		
Voltage (V)	1150	1160	1170
Nozzle-to-substrate distance (μm)	280	290	300
Viscosity (mPa·s)	35	50	65
Conductivity (μS/cm)	5	10	15

**Table 3:** Combination of SVR model parameters.

*C*	*σ*	*ε*
5	0.1	0.001
10	0.3	0.01
20	0.7	0.1
40	1	1

**Table 4:** Combination of ANN search parameters.

Number of Neurons in Hidden Layer 1	Number of Neurons in Hidden Layer 2	Number of Neurons in Hidden Layer 3
5	5	5
10	10	10
15	15	15
20	20	20

**Table 5:** Comparison of predicted and experimental results.

Sample Number	Experimental Ejection Cycle Time (ms)	Predicted Ejection Cycle Time of RFR (ms)	Relative Error (%)	Experimental Droplet Diameter (μm)	Predicted Droplet Diameter of RFR (μm)	Relative Error (%)
1	0.284 ± 0.013	0.265	−6.690	28.353 ± 0.099	29.153	2.822
2	0.876 ± 0.023	0.866	−1.142	27.555 ± 0.304	27.270	−1.034
3	0.355 ± 0.016	0.324	−8.732	22.874 ± 0.098	23.179	1.333
4	1.98 ± 0.034	2.048	3.434	29.688 ± 0.269	28.947	−2.496
5	0.556 ± 0.013	0.541	−2.698	24.469 ± 0.643	24.344	−0.511
6	1.22 ± 0.034	1.207	−1.066	24.432 ± 0.206	26.540	8.628
7	0.472 ± 0.017	0.486	2.966	26.827 ± 0.605	25.622	−4.492
8	1.224 ± 0.021	1.313	7.271	31.188 ± 0.202	30.438	−2.405
9	0.312 ± 0.017	0.296	−5.128	27.980 ± 0.360	28.630	2.323

## Data Availability

Data are contained within the article or Supplementary Material.

## Supplementary Materials

The following supporting information can be downloaded at: https://www.mdpi.com/article/10.3390/mi14030623/s1, Table S1: The compositions and properties of the liquids used in the experiments; Table S2: Experimental and predicted values of the ejection cycle; Table S3: Experimental and predicted values of the droplet diameter; Figure S1: Nozzle connection diagram, Figure S2: Variation in ejection cycle and droplet diameter with voltage; Figure S3: Variation in ejection cycle and droplet diameter with nozzle-to-substrate distance.

## References

[1] Gañán-Calvo A.M. (1997). Cone-Jet Analytical Extension of Taylor’s Electrostatic Solution and the Asymptotic Universal Scaling Laws in Electrospraying. Phys. Rev. Lett..

[2] Li K., Wang D., Wang Q., Song K., Liang J., Sun Y., Madoua M. (2018). Thermally Assisted Electrohydrodynamic Jet High-Resolution Printing of High-Molecular Weight Biopolymer 3D Structures. Macromol. Mater. Eng..

[3] Wang J., Huang R., Chen H., Qiao X., Shi X., Wang X., Cheng Y., Tan W., Tan Z. (2019). Personalized Single-Cell Encapsulation Using E-Jet 3D Printing with AC-Pulsed Modulation. Macromol. Mater. Eng..

[4] Kim K., Kim G., Lee B.R., Ji S., Kim S.Y., An B.W., Song M.H., Park J.U. (2015). High-Resolution Electrohydrodynamic Jet Printing of Small-Molecule Organic Light-Emitting Diodes. Nanoscale.

[5] Kim B.H., Onses M.S., Lim J.B., Nam S., Oh N., Kim H., Yu K.J., Lee J.W., Kim J.H., Kang S.K. (2015). High-Resolution Patterns of Quantum Dots Formed by Electrohydrodynamic Jet Printing for Light-Emitting Diodes. Nano Lett..

[6] Li Z., Jeong Y.J., Hong J., Kwon H.J., Ye H., Wang R., Choi H.H., Kong H., Hwang H., Kim S.H. (2022). Electrohydrodynamic-Jet-Printed Phthalimide-Derived Conjugated Polymers for Organic Field-Effect Transistors and Logic Gates. ACS Appl. Mater. Interfaces.

[7] Hong S., Na J.W., Lee I.S., Kim H.T., Kang B.H., Chung J., Kim H.J. (2020). Simultaneously Defined Semiconducting Channel Layer Using Electrohydrodynamic Jet Printing of a Passivation Layer for Oxide Thin-Film Transistors. ACS Appl. Mater. Interfaces.

[8] Seong B., Yoo H., Nguyen V.D., Jang Y., Ryu C., Byun D. (2014). Metal-Mesh Based Transparent Electrode on a 3-D Curved Surface by Electrohydrodynamic Jet Printing. J. Micromechanics Microengineering.

[9] Im B., Lee S.-K., Kang G., Moon J., Byun D., Cho D.-H. (2022). Electrohydrodynamic Jet Printed Silver-Grid Electrode for Transparent Raindrop Energy-Based Triboelectric Nanogenerator. Nano Energy.

[10] Vespini V., Coppola S., Todino M., Paturzo M., Bianco V., Grilli S., Ferraro P. (2016). Forward Electrohydrodynamic Inkjet Printing of Optical Microlenses on Microfluidic Devices. Lab Chip.

[11] Zhao K., Wang D., Li K., Jiang C., Wei Y., Qian J., Feng L., Du Z., Xu Z., Liang J. (2020). Drop-on-Demand Electrohydrodynamic Jet Printing of Graphene and Its Composite Microelectrode for High Performance Electrochemical Sensing. J. Electrochem. Soc..

[12] Pannico M., Musto P., Rega R., Vespini V., Grilli S., Ferraro P. (2020). Direct Printing of Gold Nanospheres from Colloidal Solutions by Pyro-Electrohydrodynamic Jet Allows Hypersensitive SERS Sensing. Appl. Surf. Sci..

[13] He L., Lu J., Han C., Liu X., Liu J., Zhang C. (2022). Electrohydrodynamic Pulling Consolidated High-Efficiency 3D Printing to Architect Unusual Self-Polarized β-PVDF Arrays for Advanced Piezoelectric Sensing. Small.

[14] Liang H., Yao R., Zhang G., Zhang X., Liang Z., Yang Y., Ning H., Zhong J., Qiu T., Peng J. (2022). A Strategy toward Realizing Narrow Line with High Electrical Conductivity by Electrohydrodynamic Printing. Membranes.

[15] Cui Z., Han Y., Huang Q., Dong J., Zhu Y. (2018). Electrohydrodynamic Printing of Silver Nanowires for Flexible and Stretchable Electronics. Nanoscale.

[16] Qin H., Wei C., Dong J., Lee Y.S. (2017). Direct Printing and Electrical Characterization of Conductive Micro-Silver Tracks by Alternating Current-Pulse Modulated Electrohydrodynamic Jet Printing. J. Manuf. Sci. Eng. Trans. ASME.

[17] An S., Lee M.W., Kim N.Y., Lee C., Al-Deyab S.S., James S.C., Yoon S.S. (2014). Effect of Viscosity, Electrical Conductivity, and Surface Tension on Direct-Current-Pulsed Drop-on-Demand Electrohydrodynamic Printing Frequency. Appl. Phys. Lett..

[18] Chen C.-H., Saville D.A., Aksay I.A. (2006). Scaling Laws for Pulsed Electrohydrodynamic Drop Formation. Appl. Phys. Lett..

[19] Choi H.K., Park J.U., Park O.O., Ferreira P.M., Georgiadis J.G., Rogers J.A. (2008). Scaling Laws for Jet Pulsations Associated with High-Resolution Electrohydrodynamic Printing. Appl. Phys. Lett..

[20] Bober D.B., Chen C.H. (2011). Pulsating Electrohydrodynamic Cone-Jets: From Choked Jet to Oscillating Cone. J. Fluid Mech..

[21] Ball A.K., Roy S.S., Kisku D.R., Murmu N.C., Coelho L. (2020). dos S. Optimization of Drop Ejection Frequency in EHD Inkjet Printing System Using an Improved Firefly Algorithm. Appl. Soft Comput..

[22] Qian L., Lan H., Zhang G. (2018). A Theoretical Model for Predicting the Feature Size Printed by Electrohydrodynamic Jet Printing. Appl. Phys. Lett..

[23] Wang Z., Wang Q., Zhang Y., Jiang Y., Xia L. (2020). Formation of Mono-Dispersed Droplets with Electric Periodic Dripping Regime in Electrohydrodynamic (EHD) Atomization. Chin. J. Chem. Eng..

[24] Collins R.T., Jones J.J., Harris M.T., Basaran O.A. (2008). Electrohydrodynamic Tip Streaming and Emission of Charged Drops from Liquidcones. Nat. Phys..

[25] Jiang L., Yu L., Premaratne P., Zhang Z., Qin H. (2021). CFD-Based Numerical Modeling to Predict the Dimensions of Printed Droplets in Electrohydrodynamic Inkjet Printing. J. Manuf. Process..

[26] Guo L., Duan Y., Huang Y., Yin Z. (2018). Experimental Study of the Influence of Ink Properties and Process Parameters on Ejection Volume in Electrohydrodynamic Jet Printing. Micromachines.

[27] Fathipour-Azar H. (2022). Polyaxial Rock Failure Criteria: Insights from Explainable and Interpretable Data-Driven Models. Rock Mech. Rock Eng..

[28] Jackulin Mahariba A., Jackulin Mahariba A.J., Jackulin Mahariba A.J., Uthra A., Rajan G.B. (2022). An Efficient Automatic Accident Detection System Using Inertial Measurement through Machine Learning Techniques for Powered Two Wheelers. Expert Syst. Appl..

[29] Liu Y., Yan W., Zhu H., Tu Y., Guan L., Tan X. (2022). Study on Bandgap Predications of ABX3-Type Perovskites by Machine Learning. Org. Electron..

[30] Li T., Yang Y., Huang J., Chen R., Wu Y., Li Z., Lin G., Liu H., Wu M. (2022). Machine Learning to Predict Post-Operative Acute Kidney Injury Stage 3 after Heart Transplantation. BMC Cardiovasc. Disord..

[31] BREIMAN L. (2001). Random Forests. Mach. Learn..

[32] Yin L., Sun Z., Gao F., Liu H. (2020). Deep Forest Regression for Short-Term Load Forecasting of Power Systems. IEEE Access.

[33] Montesinos-López O.A., Montesinos-López A., Mosqueda-Gonzalez B.A., Montesinos-López J.C., Crossa J., Ramirez N.L., Singh P., Valladares-Anguiano F.A. (2021). A Zero Altered Poisson Random Forest Model for Genomic-Enabled Prediction. G3 Genes|Genomes|Genet..

[34] Lee S., Kim J. (2020). Prediction of Nanofiltration and Reverse-Osmosis-Membrane Rejection of Organic Compounds Using Random Forest Model. J. Environ. Eng..

[35] Alabdulkarim A., Al-Rodhaan M., Tian Y., Al-Dhelaan A. (2019). A Privacy-Preserving Algorithm for Clinical Decision-Support Systems Using Random Forest. Comput. Mater. Contin..

