# Supplementary material for: Prediction of Both E-Jet Printing Ejection Cycle Time and Droplet Diameter Based on Random Forest Regression
Source: Micromachines (Basel). 2023 Mar 8;14(3):623. doi: 10.3390/mi14030623 (PMC10056634; doi:10.3390/mi14030623)
Supplement: Supplementary file 1 [file micromachines-14-00623-s001.zip › micromachines-2251093-supplementary.pdf]

Supporting Information

Prediction of Droplet Ejection Cycle and Diameter for Electrohydrodynamic Jet Printing Based on Random Forest Regression

Yuanfen Chen, Zongkun Lao, Renzhi Wang, Jinwei Li, Jingyao Gai, Hui You*
School of Mechanical Engineering, Guangxi University, Nanning, 530004, China
* Correspondence: usmlhy@iim.ac.cn (H.Y.)

Table S1 The compositions and properties of the liquids used in the experiments

Glycerol(mL)	NaCl aqueous solution(μL)	deionized water(μL)	Conductivity （μS/cm）	Density （Kg/m^3）	Viscosity （mPa·s）	Surface tension （mN/m）
5	65	1935	5	1223.9	35	66.7
5	140	1860	10	1223.9	35	66.7
5	205	1795	15	1223.9	35	66.7
5	105	1395	5	1231.6	50	67.3
5	205	1295	10	1231.6	50	67.3
5	250	1250	15	1231.6	50	67.3
5	125	1135	5	1238.9	65	66.9
5	265	985	10	1238.9	65	66.9
5	410	840	15	1238.9	65	66.9

Table S2 Experimental and predicted values of the ejection cycle

Sample number	Experimental Injection Cycle(ms)	Predicted Injection Cycle of RFR (ms)	Predicted Injection Cycle of CART (ms)	Predicted Injection Cycle of SVR (ms)	Predicted Injection Cycle of ANN (ms)
1	0.284±0.013	0.265	0.228	0.283	0.270
2	0.876±0.023	0.866	0.952	0.748	0.831
3	0.355±0.016	0.324	0.385	0.488	0.402
4	1.98±0.034	2.048	1.49	2.240	2.178
5	0.556±0.013	0.541	0.616	0.619	0.615
6	1.22±0.034	1.207	0.996	0.824	1.829
7	0.472±0.017	0.486	0.500	0.481	0.437
8	1.224±0.021	1.313	1.470	1.288	1.168
9	0.312±0.017	0.296	0.264	0.336	0.405

Table S3 Experimental and predicted values of the droplet diameter

Sample number	Experimental droplet diameter (μm)	Predicted droplet diameter of RFR (μm)	Predicted droplet diameter of CART (μm)	Predicted droplet diameter of SVR (μm)	Predicted droplet diameter of ANN (μm)
1	28.353±0.099	29.153	28.170	30.131	29.639
2	27.555±0.304	27.270	26.409	28.591	28.942
3	22.874±0.098	23.179	23.427	25.042	24.046
4	29.688±0.269	28.947	28.906	29.860	30.865
5	24.469±0.643	24.344	23.548	27.325	26.841
6	24.432±0.206	26.540	27.017	26.179	25.554
7	26.827±0.605	25.622	25.601	28.609	28.016
8	31.188±0.202	30.438	30.581	31.705	31.951
9	27.980±0.360	28.630	27.792	29.468	28.580

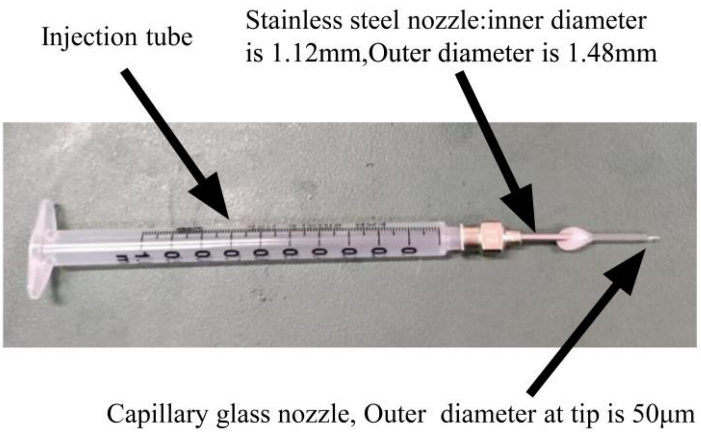

Figure S1. Nozzle connection diagram

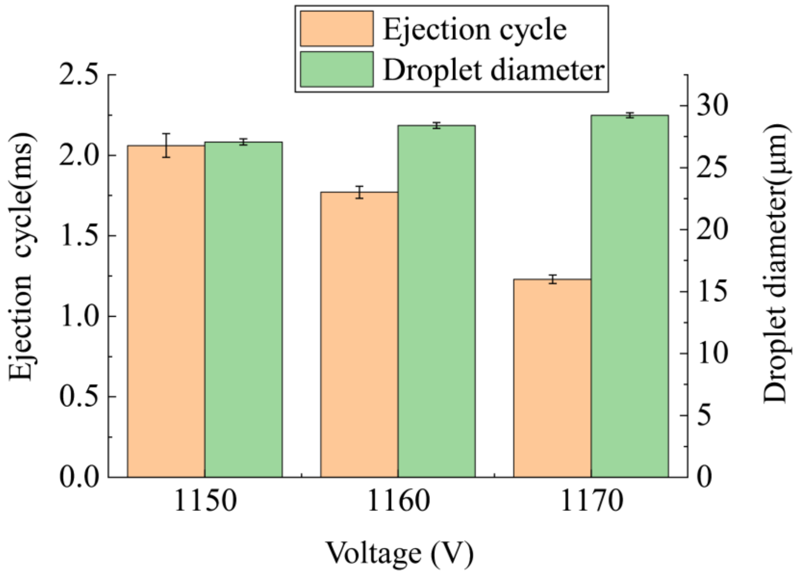

Figure S2. Variation of ejection cycle and droplet diameter with voltage(viscosity is 65 mPa·s, conductivity is 10 μS/cm, and nozzle-to-substrate distance is 300 μm).

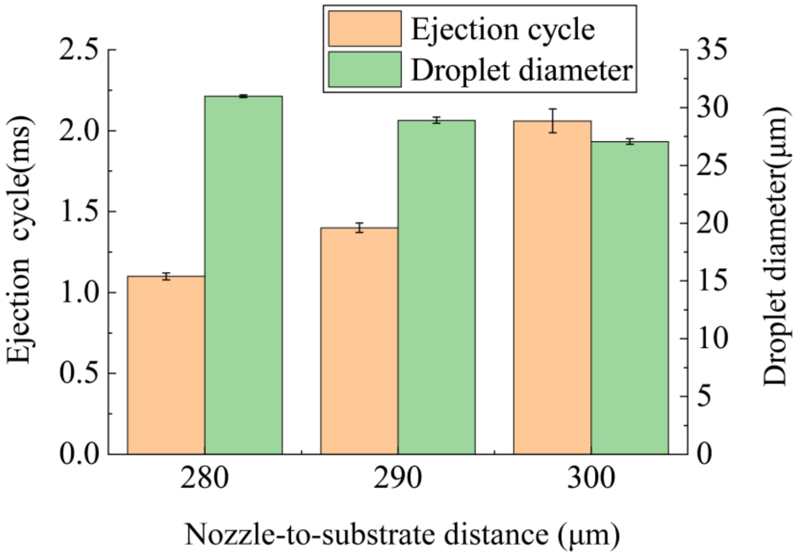

Figure S3. Variation of ejection cycle and droplet diameter with nozzle-to-substrate distance (viscosity is 65 mPa·s, conductivity is 10 μS/cm, voltage is 1150V).
